# Sarcoptic Mange in Wild Caprinae of the Alps: Could Pathology Help in Filling the Gaps in Knowledge?

**DOI:** 10.3389/fvets.2020.00193

**Published:** 2020-05-05

**Authors:** Sara Turchetto, Federica Obber, Luca Rossi, Stefano D'Amelio, Serena Cavallero, Alessandro Poli, Francesca Parisi, Paolo Lanfranchi, Nicola Ferrari, Debora Dellamaria, Carlo V. Citterio

**Affiliations:** ^1^Freelance Veterinary Pathologist, Belluno, Italy; ^2^Istituto Zooprofilattico Sperimentale delle Venezie—SCT2 Belluno—U.O. Ecopatologia, Belluno, Italy; ^3^Dipartimento di Scienze Veterinarie, Università degli Studi di Torino, Grugliasco, Italy; ^4^Dipartimento di Sanità Pubblica e Malattie Infettive, Università di Roma “La Sapienza”, Rome, Italy; ^5^Dipartimento di Scienze Veterinarie, Università di Pisa, Pisa, Italy; ^6^Dipartimento di Medicina Veterinaria, Università degli Studi di Milano, Lodi, Italy; ^7^Istituto Zooprofilattico Sperimentale delle Venezie—SCT5 Trento, Trento, Italy

**Keywords:** *Sarcoptes scabiei*, caprinae, pathology, sarcoptic mange, immune response, hypersensitivity

## Abstract

Sarcoptic mange represents the most severe disease for wild Caprinae individuals and populations in Europe, raising concerns for both conservation and management of these ungulates. To date, this disease has been investigated in different wild caprine species and under many different perspectives including diagnostics, epidemiology, impact on the host populations, and genetics of both hosts and parasite, with the aim to disentangle the host–*Sarcoptes scabiei* relationship. Notwithstanding, uncertainty remains and basic questions still need an answer. Among these are the effect of immune responses on mange severity at an individual level, the main drivers in host–parasite interactions for different clinical outcomes, and the role of the immune response in determining the shift from epidemic to endemic cycle. A deeper approach to the pathology of this disease seems therefore advisable, all the more reason considering that immune response to *S. scabiei* in wild Caprinae, generally classified as a hypersensitivity, remains poorly understood. In this paper, we reviewed the pathological features associated to sarcoptic mange in wildlife, exploring different kinds of hypersensitivity and outcomes, with the objective of highlighting the major drivers in the different responses to this disease at an individual level and proposing some key topics for future research, with a particular attention to Alps-dwelling wild caprines.

## Introduction

**Figure 1:**
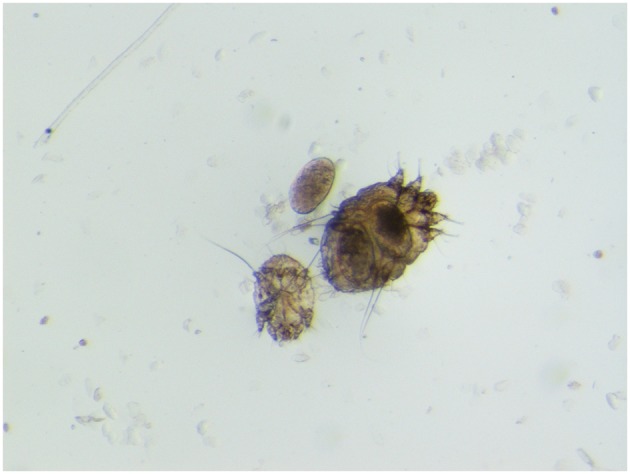
*Sarcoptes scabiei* var. *rupicaprae* (stereomicroscope 60×) from a mangy northern chamois (*Rupicapra r. rupicapra*) in northeast Italy.

Sarcoptic mange (or scabies) due to the burrowing mite *Sarcoptes scabiei* is reported worldwide in domestic and wild mammals, in the latter often representing a threat to conservation due to evident effects on population dynamics. Some varieties of this mite have been described as being able to infect a specific range of zoologically related hosts. Mites infecting the Northern chamois (*Rupicapra r. rupicapra*), a scabies sensitive caprine (Fam. Caprinae) on which this paper is mainly focused, are referred to as *S. scabiei* var. *rupicaprae* (Figure 1). Besides chamois, they are experimentally and/or naturally cross-transmissible to the Alpine ibex (*Capra ibex*) and the domestic goat, while sporadically to domestic sheep, mouflon (*Ovis aries musimon*), roe deer (*Capreolus capreolus*), and red deer (*Cervus elaphus*) (1, 2).

Sarcoptic mange is probably the most severe disease affecting wild Caprinae in Europe (3), as evidenced by the epizootics affecting the Spanish ibex (*Capra pyrenaica*) in the southeast of Spain (4, 5), and the northern chamois and Alpine ibex in the Eastern Alps in Italy, Austria, Germany, and Slovenia (6–8). Moreover, sarcoptic mange has been severely affecting two other wild Caprinae hosts in Southern Europe, namely the western subspecies of the southern chamois [*Rupicapra pyrenaica parva;* (9)] and the exotic Barbary sheep [*Ammotragus lervia*; (10)].

Besides serious consequences for endangered taxa, where losing even a few individuals can be critical for conservation (11), this disease may also raise concern over the wildlife-based economy, due to the setbacks of population downturns. This is well-represented in the Eastern Alps, where recent mange outbreaks have caused doubt over 30 years of commendable conservation efforts of the locally fragile Alpine ibex (an easy to spot symbol for mountain hikers), and a sympatric game, the alpine subspecies of the northern chamois, currently ranked “Least concern” by IUCN due to its wide and abundant distribution, has also experienced significant population crashes, which have led to a sharp reduction and even the banning of any harvest in affected game management units (3, 7).

Due to its impact on wild caprine populations and the related conservation issues, sarcoptic mange has been investigated from a range of perspectives. In Europe, research has been performed on epidemiology [e.g., (1, 3–7, 9, 10)], including spatial epidemiology (12), immunodiagnosis (13, 14), experimental infection (15–18), mite genetics (19, 20), and host genetics. Concerning this latter topic, special emphasis has been given to the major histocompatibility complex (MHC), deemed crucial to cope with infectious agents including *S. scabiei* and other pathogens (21–23). Studies on host genetics are believed to be of particular interest also due to the typical pattern of sarcoptic mange in these species, where the first epidemic peak in naïve populations may exert a significant demographic impact (up to more than 80% reduction of the population size), followed by less severe outbreaks (“waves”) at 10- to 15-years intervals with a low mortality rate rarely exceeding 25% (3). Such a cyclic pattern could be suggestive of dynamics based on the presence of an advantageous genetic background in those animals surviving the exposition, determining a selection of individuals with higher genetic resistance (24), which, combined with the lowered population density, could explain the minor waves after the introduction of the pathogen. In addition, balancing selection may favor, at the same time, heterozygosity and the retaining of rare alleles. To investigate this hypothesis, adaptive processes need to be explored, and for this purpose, MHC genes are excellent candidates (21), representing a promising research field to explore the Caprinae-*S. scabiei* relationship. In fact, intracellular and extracellular pathogens trigger a strong immune response in such species, and the study of the genetic background of the MHC molecules may assist the identification of resistance-associated or rare alleles, which may be involved in the survival of individuals with peculiar polymorphisms in these genetic regions. Under this hypothesis, however, it would be expected that different genetic backgrounds are reflected in different kinds and/or degrees of immune response and consequent pathological pictures. Indeed, pathology should precede and inform studies on the host–parasite relationship and coevolution, including genetics, but in the case of sarcoptic mange, the investigation path is not so linear. In our opinion, however, pathology can give a remarkable contribution to the study of mange ecology and epidemiology in wildlife, and in particular in Caprinae, thus helping to fill the longtime gap in knowledge in Europe (25) resumed by the research questions in Box 1 (26).

Box 1 **Gaps in knowledge to fill concerning sarcoptic mange in wildlife in Europe (26).**

**Table:** 

Which dynamics are actually involved in the shift from epidemic to endemic cycle of sarcoptic mange in wildlife populations?
How do factors such as coinfections, health condition, and genetic background influence mange infestation in wildlife?
How can mortality due to mange be properly assessed and differentiated from other causes of death?
Is sarcoptic mange a threat for biodiversity conservation? Which species or populations should be considered for intervention?

In recent years, studies on the pathology of sarcoptic mange in European wild ruminants have been focused on the impact of this disease on individuals and populations (18, 27) or on early diagnostics (28), while less attention has been paid to characterizing mange lesions and ranking them according to severity and chronology of the pathological process (29, 30). Assuming that traits of genetic resistance may underlie diverse immune response type and intensity among different individuals (31), a fine-tuned pathological description could help answer some of the questions in Box 1, e.g., by correlating selected macroscopic and histopathological frames to specific epidemiological phases (epidemic vs. endemic), coinfections, and genetic backgrounds. Hence, in this paper, we have explored the pathology of sarcoptic mange in wildlife, with the objective of highlighting the major drivers in the range of host responses to the disease and proposing some key issues for future research at both an individual and a population level, with a particular attention to Alps-dwelling wild Caprinae.

## Hypersensitivity as the Basis of Tissue Damage in Sarcoptic Mange

Ectoparasites are known to cause a range of unfavorable effects, as damage to skin and predisposition to secondary infection. Moreover, they can serve as vectors of infectious agents such as viruses, bacteria, spirochetae, rickettsiae, and protozoans. The cutaneous reaction to parasites, often mediated by immune mechanisms, varies with parasite abundance, location, feeding habits, and host immune recognition (32).

Diseases associated with excessive or aberrant immune responses are classified as either hypersensitive (allergy) or autoimmune. Microscopically, evidence in all studied host species indicates that sarcoptic mange is a complex hypersensitivity reaction to various mite antigens, as proteins in the cuticle and feces, producing most of the clinical features (33–37). Actually, the mechanisms of tissue injury in hypersensitivity reactions are the same as the effector mechanisms of defense against infectious pathogens, but in hypersensitivity reactions, they are poorly controlled, excessive, or misdirected (31). Hypersensitivity can be classified into four basic immune reaction types (I, II, III, and IV), which mediate the tissue damage in response to normally harmless foreign compounds (e.g., antiserum, pollen, insect venoms). In the skin, hypersensitivity in most cases is the result of either type I or type IV reactions, or comes from a combination of two or more of the four reaction types (32).

Type I hypersensitivity is mediated by the reaction between a foreign antigen and a specific antibody (usually IgE), enabling the release of pharmacologically active substances as histamine, chemotactic factors for eosinophils and neutrophils, and cytokines by mast cells and basophils. It can be either systemic (anaphylaxis), or localized to the skin, or both and include, as an example, atopic dermatitis, urticaria, angioedema, or hypersensitivity to bites of insects and mites as *Sarcoptes* sp. This reaction is characterized by mast cell degranulation, capillary dilation, edema, and infiltrates of eosinophils (32).

On the other hand, type IV reactions are mediated by antigen-specific effector T lymphocytes, which include sensitized T-helper lymphocytes (Th1 or Th2). Th1-mediated reactions develop after contact with a specific persistent antigen and cause the recruitment of other lymphocytes and macrophages to eliminate the targeted antigen. In Th2-mediated reactions, contact with soluble antigen bound to MHC class II (MHC II) causes inflammatory responses dominated by eosinophils (32).

## Sarcoptic Mange From Macroscopic Lesions to Microscopic Features

**Figure 2:**
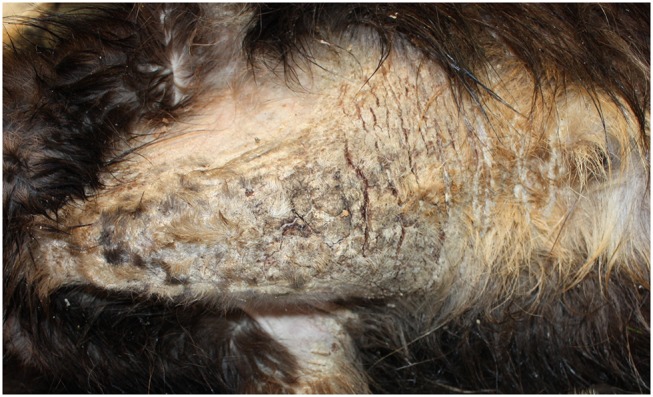
Severe sarcoptic mange in a northern chamois *Rupicapra r. rupicapra* (NE Italy). Particularly of the chest. Macroscopically, lesions are characterized by severe diffuse chronic hyperplastic and hyperkeratotic dermatitis with alopecia and crusting.

Itching is a frequent symptom in cutaneous hypersensitivity reactions and represents the first clinical feature also for sarcoptic mange. An erythematous maculopapular eruption with alopecia, hyperkeratosis, and the formation of crusts is accompanied by self-traumatic excoriations, leading to secondary pyoderma. In general, lesions first affect the margins of the pinnae, followed by ventral truncal abdomen, chest, lateral elbows, and face involvement (38). However, localization of the early lesions also depends on the skin area first exposed to the infection. Extensive alopecia, lichenification, and hyperpigmentation may be seen in chronic cases (29) (Figure 2).

Studies have shown that the response to mange is different among host species. For instance, dogs, humans, and rabbits acquire full immunity, while in the red fox, immune response appears to be less effective at restricting infection due to lack of memory T-cells after initial phases. Additionally, the immune response can be uneven within the same species according to the sex of the individual or its previous exposure status (26). Field evidence suggests, however, that such an unevenness could also depend on other still unknown factors: namely, in both northern chamois and Alpine ibex, it is renowned that the impact of mange has been higher in some populations than in others. For example, the impact of *S. scabiei* on the demography of the Alpine ibex colonies in northeastern Italian Alps has ranged from extinction and dramatic crashes to relatively low population reduction, so that ibex individuals from the latter are used *ex juvantibus* for the restocking of the former (39).

**Figure 3:**
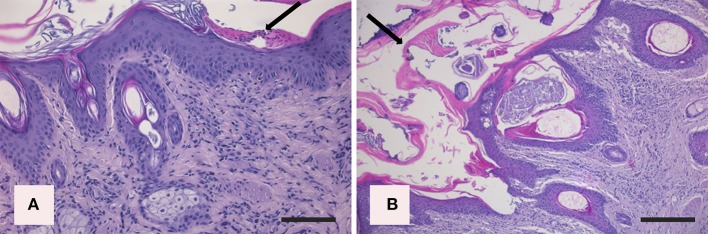
Histopathologic lesions in northern chamois (*Rupicapra r. rupicapra*) affected by sarcoptic mange. The following pictures are further evidenced by black arrows: **(A)** Moderate, diffuse, irregular epidermal hyperplasia with neutrophilic serocellular crust in grade 2 sarcoptic mange (hematoxylin and eosin, bar = 300 μm). **(B)** Severe, diffuse, orthokeratotic to compact hyperkeratosis with thick neutrophilic serocellular crusts in a grade 3 sarcoptic mange, which correlate with the increased number of mites (hematoxylin and eosin, bar = 300 μm). Further details available in Salvadori et al. (40).

**Figure 4:**
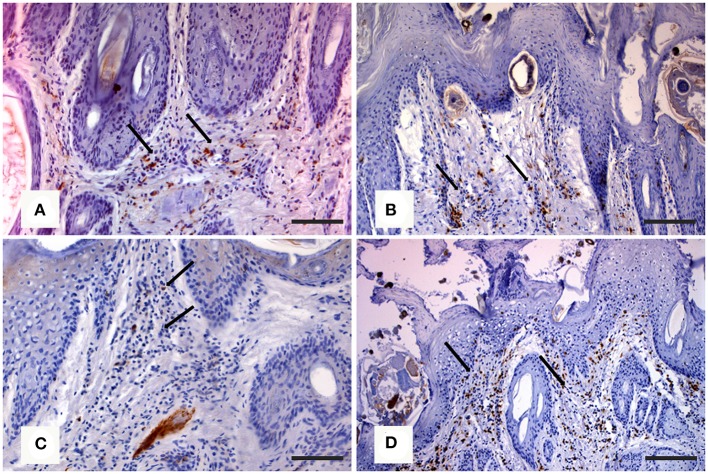
Immunohistochemical staining of skin in northern chamois (*Rupicapra r. rupicapra)* affected by sarcoptic mange. The following pictures are further evidenced by black arrows: **(A)** Diffuse macrophages infiltration in the superficial dermis in grade 2 sarcoptic mange (IHC, anti CD68 antibody, DAB chromogen, and hematoxylin counterstain, bar = 300 μm). **(B)** Numerous T lymphocytes in the superficial dermis of a chamois with grade 2 sarcoptic mange (IHC, anti CD3 antibody, DAB chromogen, and hematoxylin counterstain, bar = 300 μm). **(C)** Increased B lymphocytes infiltration in the superficial dermis in a grade 3 sarcoptic mange (IHC, anti CD79a antibody, DAB chromogen, and hematoxylin counterstain, bar = 300 μm). **(D)** Scattered T lymphocytes among B cells in a grade 3 lesion (IHC, anti CD3 antibody, DAB chromogen, and hematoxylin counterstain, bar = 300 μm). Further details available in Salvadori et al. (40).

*In the northern chamois*, histological skin lesions due to sarcoptic mange have been recently classified in three grades (40) according to crust thickness, alopecia, mite counts, infiltration of eosinophils, lymphocytes, and mast cells. Grades 1 and 2 are characterized by slight to moderate crust formation and a low number of mites, while grade 3 represents the most severe stage with crust thickness of more than 3.5 mm (Figures 3A,B), which correlates with a very high number of mites and severe parakeratosis with diffuse bacterial aggregates. The presence of numerous and thick serocellular crusts is apparently a mechanism destroying mites or inhibiting their burrowing into the skin, as these crusts contain specific antibodies and other toxic components leading to mite intoxication (18). In all stages, an increased number of eosinophils, mast cells, T and B lymphocytes, and macrophages are associated with diffuse severe epidermal hyperplasia, suggesting both type I and type IV hypersensitivity. Analogously to human scabies, grades 1 and 2 could be suggestive of skin immune response to *S. scabiei* with adequate presence of macrophages (Figure 4A), T lymphocytes (Figure 4B), and eosinophils that allow parasite control through a reduction in mite numbers and limitation of lesions. Moreover, numbers of eosinophils in the dermis are correlated with the density of mites, suggesting that recruitment of eosinophils is influenced by the parasites or their products (41). In contrast, grade 3 lesions seem to be characterized by a significant increase of B lymphocytes (Figure 4C) and a decrease in eosinophils without a significant increase in macrophages and T lymphocytes (Figure 4D) (40). Infiltration of Langerhans cells is also observed in the epidermis of mangy chamois, and it is assumed that epidermal dendritic cells with captured sarcoptic antigen migrate through lymphatic vessels into the draining regional lymph nodes. Here, they present it to naïve T cells and induce their differentiation into Th1 and Th2. Th1-type reactions tend to produce proinflammatory and microbicidal responses, counteracted with Th2-type reactions including molecules promoting IgE production (IL-4, IL-5, IL-10, and IL-13) and eosinophilic responses in allergies, and also an anti-inflammatory response (42). Antigen presentation is likely to be increased in the dermis of chamois with sarcoptic mange, as suggested by the increased number of T and B lymphocytes, macrophages, and eosinophils in grade 3 lesions compared to grade 1: in particular, the increased B lymphocyte and eosinophil numbers in grade 3 compared to grade 1 appears the most significant (40). Grades 1 and 2 lesions with slight to moderate crust formation and a lower abundance of mites could therefore be thought of as the initial stages, while grade 3 could be interpreted as the following, generalized, severe stage. As in human scabies, grades 1 and 2 could be suggestive of adequate skin immune response to *S. scabiei*, allowing parasite control and limitation of lesions. In contrast, grade 3 lesions, characterized by significant B lymphocyte and eosinophils increase, could suggest a Th2 weighted imbalance, involving an IgE-driven nonprotective Th2 response, that could affect the control and reduction of the mite burden particularly in sequential infestations (40). The role of Langerhans cells in the pathogenesis of chamois mange, however, remains obscure, and research is needed on the distributional changes of these cells in response to infection (29).

**Figure 5:**
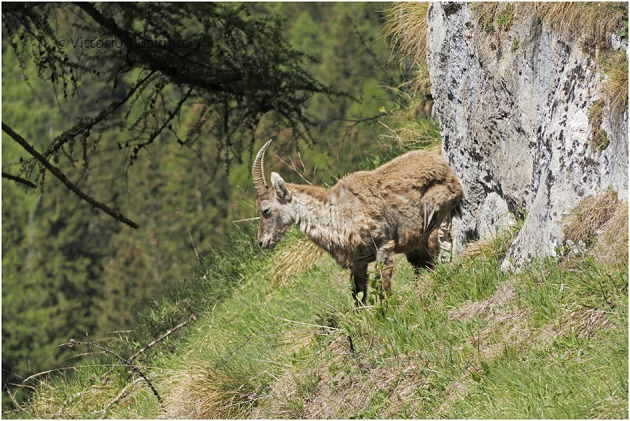
Severe sarcoptic mange in an Alpine ibex (*Capra ibex*) from NE Italy. Macroscopically, lesions are characterized by severe, multifocal to coalescing, chronic hyperplastic dermatitis with alopecia and lichenification. Courtesy of Mr. Vittorio Fusinato.

For *Alpine ibex*, although gross lesions in mangy animals are often dramatic (Figure 5), no detailed histological data are available at the moment. The histological picture has been instead described in the *Spanish ibex* (18), where the skin lesions observed closely match the classical picture of wild and domestic animals affected by sarcoptic mange. The cellular response is largely dominated by mononuclear cells and marginally by eosinophils and mast cells, which may explain the presence of less obvious marked hyperkeratosis. Similarly to chamois, ibex skin lesions are compatible with a combined type I and type IV hypersensitivity reaction. Type I hypersensitivity is characterized by cytotoxicity signs (e.g., spongiosis), positively correlated with the number of inflammatory cells. Subsequently, these signs decrease along with the cellular infiltrate, which suggests type IV hypersensitivity. As in all other species, the number of plasma cells in mangy ibex is low, which may be linked to a reduced role of the humoral response. Moreover, also in ibex, the overexpression of eosinophils, mast cells, and neutrophils is associated with a detrimental and non-protective response, unable to control the infection.

**Figure 6:**
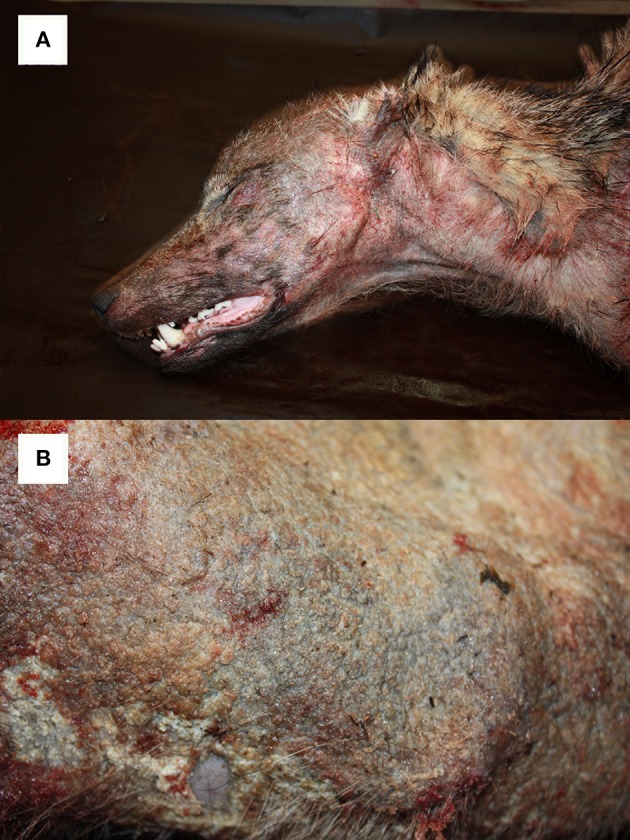
Alopecic form of sarcoptic mange affecting a young (1–2 years) female wolf (*Canis lupus*) from NE Italy. Particularly of the head **(A)** and the trunk **(B)**.

For comparison among different taxa, it is worthwhile reporting the skin features of European mangy *carnivores*. In *red foxes* (*Vulpes vulpes*) infected with *S. scabiei* var*. vulpes*, skin lesions associated with necropsy findings have also been classified in three types (43). In this host after an early stage A, characterized by focal-extensive skin lesions, thin crusts, mild to moderate alopecia, few mites, and numerous eosinophils, the subject either enters stage B (hyperkeratotic, “fatal” form) with generalized skin lesions, thick crusts, and increased mite number, lymphocytes, and mast cells, leading in most cases to death; or stage C (alopecic, “healing” form), characterized by focal severe alopecia, hyperpigmentation and lichenification, absence of mites, and mixed cell infiltration, leading to possible survival. As both mast cell and mite counts increase in foxes showing type B lesions compared to type A, unknown extrinsic or intrinsic factors, such as a dysregulation of the immune system, may result in impairment of the eosinophilic function, affecting the host's ability to control mite infestation and thus playing an important role in determining the disease course. The scarce number of mites detected in the skin of red foxes with the alopecic form contrasts with the numerous mites observed in individuals showing the hyperkeratotic form, and it is interesting to point out that the latter generally represents the typical picture in mountain caprines. A further interesting comparison can be made with another carnivore, the *wolf* (*Canis lupus*) where, in contrast to the chamois, the abundance of mites decreases in the lesions as the hypersensitivity reaction progresses (44). In animals showing the alopecic form, neutrophils establish the inflammatory infiltrate: although neutrophils can be related to secondary infections or skin damage, the oxidation burst of these cells may be important in eliminating mites (34). Both macroscopical and microscopical skin lesions by sarcoptic mange in wolves suggest a prevalence of the alopecic form (Figures 6A,B) rather than the hyperkeratotic one; the coincidence in wolves of this presentation with the apparent higher ability of this species to control the parasite (45) could suggest that the immune response in the alopecic form represents a more effective defense mechanism against *S. scabiei* (44). A higher efficiency of the wolf immune system in controlling the mite proliferation could also allow this host to reduce the number of mite generations on itself. Actually, the detection in a wolf of both “ungulate-like” and “red fox-like” mites suggests that repeated infestations are possible with mites from any of the sympatric species this canid may get in contact with. These features may inhibit host adaptation, thus facilitating gene flow among mite populations and preventing genetic selection, finally determining the high variability reported in *C. lupus*–derived mites. In the case of the wolf, its wide spatial range and the large number of species this canid can potentially contact (both as a predator and as a scavenger) are additional subjects favoring repeated infestations by genetically different mites from different host species (20).

**Figure 7:**
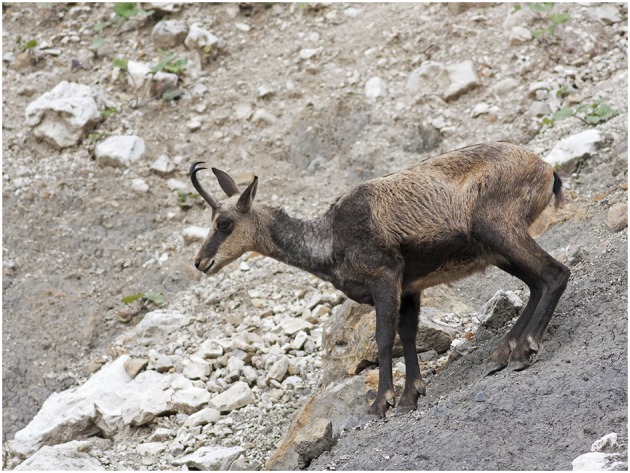
A follow-up in the field led to the hypothesis that the northern chamois in this picture (NE Italy) was healing from sarcoptic mange. This adult female is characterized by moderate, focally extensive, chronic hyperplastic dermatitis with alopecia and lichenification. Monitoring healing processes in wild animals are challenging due to difficulties in regularly observing specific individuals. At the same time, capturing or culling of such individuals should be absolutely avoided, since under the hypothesis of a genetic resistance to *S. scabiei*, they could represent an essential resource for the population. Healing of Alpine ibex individuals have also been reported anecdotally. Courtesy of Mr. Vittorio Fusinato.

An important remark is finally related to lethality, since for Spanish ibex (46) and red fox (43), there is evidence that some free-ranging individuals are able to heal from sarcoptic mange, whereas for northern chamois and Alpine ibex, evidence of recovery remains anecdotal (Figure 7).

## Perspectives in the Pathology of Sarcoptic Mange in Alpine Wild Caprinae

Although uncertainty remains for mange pathology in wild Caprinae, steps to understand immune responses to *S. scabiei* [(26), Box 2] are being made, and therefore prospects for a deeper understanding are positive. Key areas that we consider research priorities include the following:

Box 2 **Research questions about immune response to *S. scabiei* in wildlife (26).**

**Table:** 

How can the effect of immunological responses on mange severity be studied in wildlife?
Which factors are the primary drivers in host–parasite interactions for both positive and negative clinical outcomes of mange in wildlife?

### Role of the Oxidative Burst

As seen in previous studies, numerous mononuclear cells, mast cells, and plasma cells may play a key role in protective immunity. Observation about preferential infiltration of neutrophils, due not only to the presence of mites but also to potential secondary bacterial infections or excoriations, and evaluation of the progression or regression of the disease may inform on the oxidative burst role in effective protection.

### Role of Different Types and Degrees of Hypersensitivity

Under field conditions, evaluating skin lesions in chamois/ibex from different epidemiological scenarios (e.g., individuals from scabies naïve vs. endemically infected populations) could help to elucidate inflammatory responses to the first and subsequent infestations. Experimentally, identifying the subpopulations of mononuclear cells in scabietic lesions during the sensitization and challenge infection phases, and characterizing the timing and intensity of the immunological response during the challenge phase, could help to elucidate their role and the mechanism for the immune response in these hosts (34).

### Role of the Intensity of the Immunologic Reaction

Skin biopsy details of inflammatory response to scabies mite antigens may inform about variability in severity of clinical signs, development of asymptomatic carriers, and decrease in mite numbers as the disease progresses. Individuals with compromised immune system (due to coexistent immunosuppressive disease and/or poor condition) may harbor much larger populations of mites. Similarly, high mite counts associated to severe clinical presentation, including the notorious crusted (or Norwegian) scabies (https://www.cdc.gov/parasites/scabies/disease.html), are seen most commonly in human patients who are immunocompromised due to many causes, among which the acquired immunodeficiency syndrome (AIDS) (47). It should be noted, however, that although variability of spongiosis and inflammation in the immediate vicinity of individual mites is postulated to be indicative of the stage of the inflammatory reaction to these parasites, histological features may vary depending upon the fortuitous presence of mites in the histological sections, as observed in dogs (48). Therefore, comparison between histological pictures among individuals and/or species should include a representative number of sections both including and not including mites.

### Role of Langerhans Cells

Distributional studies in humans have shown a preference of mites for sparsely haired skin, resulting in a predominantly ventral truncal distribution of lesions and relatively spare on the dorsum of the trunk, that could be explained by the rarity of epidermal Langerhans cells in certain regions, e.g., neck and shoulder (49). Moreover, experimental manipulation with UV radiation clearly showed abrogation of epidermal Langerhans cells in mice (50), which could explain the beginning of sensitization in certain parts of human and animal skin depending on the frequency and morphological identity of Langerhans cells, unevenly distributed (51, 52). The implementation of an immunohistochemical study on skin biopsies from healthy and mangy chamois could help to evaluate the distributional changes of these cells in the body regions and the reflections of these variations on the disease.

### Role of the MHC of the Host

It is accepted that the evaluation of genetic polymorphisms in chamois, in particular of the MHC II molecules specifically involved in reactions against extracellular pathogens, may greatly contribute to estimating the adaptive potential of populations, as well as to identifying alleles potentially related to susceptibility and resistance. Studies that combine pathology and genetics could therefore be useful to investigate genetic diversity in infected individuals, and Northern alpine chamois represents a suitable model for such studies. However, observations and data collection should be undertaken over time by the comparison between MHC class II sampled under different conditions (e.g., healthy chamois before the first epidemic wave; mangy and healthy chamois during the first outbreak; survived and younger chamois after the first and during the second epidemic wave). In fact, only a consistent and longitudinal sample could be representative to clarify whether some animals are genetically prone to allergic reactions against the mite, as well as if others are actually protected from the disease. A comparable study design investigated the chamois population dynamics in relation to different exposure levels to several pathogens, including pneumonia, Pestivirus, and sarcoptic mange (22, 53, 54). The study of MHC class II polymorphisms revealed genetic drift and balancing selection in response to the diseases: high polymorphisms and numbers of alleles were observed in individuals who survived pneumonia outbreaks, even with a high mortality rate. Similarly, genetic polymorphisms of DRB1 genes of chamois populations with different epidemiological pictures of sarcoptic mange were monitored for seven years, revealing (i) the presence of few alleles at a very high frequency in all populations, most likely selected as favorable; (ii) the presence of a higher than expected level of polymorphisms; and (iii) several alleles at low frequency observed only in populations with peculiar epidemiological scenarios (endemic, epidemics, healthy populations).

In conclusion, pathology could represent a significant clue in understanding the relationships between host and *S. scabiei*, particularly as part of the “Sarcoptes-World Molecular Network” (55, 56), aimed at harmonizing *Sarcoptes* epidemiology, diagnosis, treatment, and molecular studies from all over the world. Undoubtedly, the establishment of a similar framework focused on pathology and immune response to this parasite, besides improving the knowledge on this disease, could inform wildlife management and, in a perspective of comparative pathology, contribute to mitigation of sarcoptic mange in domestic animals and humans.

## Author Contributions

ST and CC conceived the paper. ST, LR, SC, SD'A, AP, FP, FO, DD, NF, PL, and CC wrote the manuscript. All authors contributed to manuscript revision, read, and approved the submitted version.

## Conflict of Interest

The authors declare that the research was conducted in the absence of any commercial or financial relationships that could be construed as a potential conflict of interest.

## References

[1] Alasaad S Oleaga A Casais R Rossi L Molinar Min A Soriguer RC. Temporal stability in the genetic structure of Sarcoptes scabiei under the host-taxon law: empirical evidences from wildlife-derived Sarcoptes mite in Asturias, Spain. Parasit. Vect. (2011) 4:151–7. 10.1186/1756-3305-4-151 21794141 PMC3160406

[2] Kutzer E Zur epidemiologie de Sarcoptes-raeude. Angew Parasitol. (1996) 7:241–8.

[3] Rossi L Tizzani P Rambozzi L Moroni B Meneguz PG. Sanitary emergencies at the wild/domestic caprines interface in Europe. Animals. (2019) 9:922. 10.3390/ani9110922 31694211 PMC6912786

[4] Pérez JM Ruiz-Martínez I Granados JE Soriguer RC Fandos P The dynamics of sarcoptic mange in the ibex population of Sierra Nevada in Spain – influence of climatic factors. J Wildlife Res. (1997) 2:86–9.

[5] León-Vizcaíno L Ruíz de Ybáñez MR Cubero MJ Ortíz MJ Espinosa J Pérez L. Sarcoptic mange in spanish ibex from Spain. J Wildl Dis. (1999) 35:647–59. 10.7589/0090-3558-35.4.647 10574523

[6] Rossi L Meneguz P De Martin P Rodolfi M. The epizootiology of sarcoptic mange in chamois Rupicapra rupicapra from the Italian Eastern Alps. Parassitologia. (1995) 37:233–40. 8778664

[7] Rossi L Fraquelli C Vesco U Permunian R Sommavilla GM Carmignola G Descriptive epidemiology of a scabies epidemic in chamois in the Dolomite alps, Italy. Eur J Wildl Res. (2007) 53:131–41. 10.1007/s10344-006-0067-x

[8] Schaschl H Gamsräude. Wien: österreichischer Jagd- und Fischerei-verlag Vienna (2003). p. 160.

[9] Fernández-Morán J Gómez S Ballesteros F Quirós P Benito JL Feliu C Epizootiology sarcoptic mange in a population of cantabrian chamois Rupicapra pyrenaica parva in northwestern Spain. Vet Parasitol. (1997) 115:163–71. 10.1016/S0304-4017(97)00061-7 9477502

[10] Gonzalez-Candela M Leon Vizcaìno L Cubero-Pablo MJ. Population effects of sarcoptic mange in Barbary sheep (*Ammotragus lervia*) from Sierra Espuna Regional Park, Spain. J Wildl Dis. (2004) 40:456–65. 10.7589/0090-3558-40.3.456 15465713

[11] Pence DB Ueckermann E Sarcoptic mange in wildlife. Revue Scient Techn. (2002) 21:385–98. 10.20506/rst.21.2.1335 11974622

[12] Turchetto S Obber F Permunian R Lorenzetto M Ferr è N Stancampiono L Spatial and temporal explorative analysis of sarcoptic mange in Alpine chamois (Rupicapra r. rupicapra). Hystrix Ital J Mammal. (2014) 25, 25–30. 10.4404/hystrix-25.1-9460

[13] Rambozzi L Menzano A Lavin S Rossi L. Biotin-avidin amplified ELISA for detection of antibodies to Sarcoptes scabiei in chamois (Rupicapra spp.). Vet Res. (2004) 35:701–8. 10.1051/vetres:2004039 15535959

[14] Raez-Bravo A Granados JE Serrano E Dellamaria D Casais R Rossi L. Evaluation of three enzyme-linked immunosorbent assays for sarcoptic mange diagnosis and assessment in the Iberian ibex, Capra pyrenaica. Parasit Vect. (2016) 9:558. 10.1186/s13071-016-1843-4 27769278 PMC5073795

[15] Lavin S Ruiz-Bascaran M Marco I Fondevila MD Ramis AJ. Experimental infection of chamois (*Rupicapra pyrenaica parva*) with Sarcoptes scabiei derived from naturally infected goats. J. Vet. Med. B. (2000) 47:693–9. 10.1046/j.1439-0450.2000.00406.x 11244870

[16] Menzano A Rambozzi L Molinar Min A Rossi L Experimental infection of chamois with Sarcoptes scabiei. In: Mange and myiasis of livestock. Proceedings of Workshop EU-COST Action 833. Toulouse (2002). p. 60–4. Available online at: http://op.europa.eu/en/publication-detail/-/publication/f6b03401-1939-4aed-a24a-5a5062e9d3e7/language-en/format-PDF/source-124957428

[17] Sarasa M Rambozzi L Rossi L Meneguz PG Serrano E Granados JE. Sarcoptes scabiei: specific immune response to sarcoptic mange in the Iberian ibex Capra pyrenaica depends on previous exposure and sex. Exp Parasitol. (2010) 124:265–71. 10.1016/j.exppara.2009.10.008 19857492

[18] Espinosa J Ráez-Bravo A López-Olvera JR Pérez JM Lavín S Tvarijonaviciute A. Histopathology, microbiology and the inflammatory process associated with Sarcoptes scabiei infection in the Iberian ibex, Capra pyrenaica. Paras Vec. (2017) 10:596–606. 10.1186/s13071-017-2542-5 29202802 PMC5715492

[19] Rasero R Rossi L Soglia D Maione S Sacchi P Rambozzi L Host taxon-derived Sarcoptes mite in European wild animals revealed by microsatellite markers. Biol Conserv. (2010) 143:1269–77. 10.1016/j.biocon.2010.03.001

[20] Oleaga A Alasaad S Rossi L Casais R Vicente J Maionee S. Genetic epidemiology of Sarcoptes scabiei in the Iberian wolf in Asturias, Spain. Vet Parasitol. (2013) 196:453–9. 10.1016/j.vetpar.2013.04.016 23664709

[21] Mona S Crestanello B Bankhead-Dronnet S Pecchioli E Ingrosso S D'Amelio S. Disentangling the effects of recombination, selection, and demographyon the genetic variation at a major histocompatibility complex class II gene in the alpine chamois. Mol Ecol. (2008) 17:4053–67. 10.1111/j.1365-294X.2008.03892.x 19238706

[22] Cavallero S Marco I Lavín S D'Amelio S López-Olvera JR. Polymorphisms at MHC class II DRB1 exon 2 locus in Pyrenean chamois (Rupicapra pyrenaica pyrenaica). Infect Genet Evol. (2012) 12:1020–6. 10.1016/j.meegid.2012.02.017 22425496

[23] Schaschl H Suchentrunk F. L., Morris D, Slimen HB, Smith S, et al. (2012). Sex-specific selection for MHC variability in Alpine chamois. BMC Evol Biol 12:20. 10.1186/1471-2148-12-20 22335968 PMC3340304

[24] Guberti V Zamboni L Can the host resistance hypothesis explain the cyclic patterns observed in *Sarcoptes scabiei in chamois (Rupicapra rupicapra)*? Parassitologia. (2000) 42(Suppl. 1):72.

[25] Guberti V Rossi L The wild mammals/parasite relationship: a review of the Italian literature 1970-1994. Suppl Ricerche Biol Selvagg. (1996) 17–26.

[26] Astorga F Carver S Almberg ES Sousa GR Wingfield K Niedringhaus KD. International meeting on sarcoptic mange in wildlife, June 2018, Blacksburg, Virginia, USA. Paras Vect. (2018) 11:449. 10.1186/s13071-018-3015-1 30075742 PMC6090813

[27] Espinosa J Granados JE Cano-Manuel FJ López-Olvera JR Ráez-Bravo A Romero D. Sarcoptes scabiei alters follicular dynamics in female Iberian ibex through a reduction in body weight. Vet Parasitol. (2017) 243:151–6. 10.1016/j.vetpar.2017.06.022 28807285

[28] Rahman MM Lecchi C Fraquelli C Sartorelli P Ceciliani F. Acute phase protein response in Alpine ibex with sarcoptic mange. Vet Parasitol. (2010) 168:293–8. 10.1016/j.vetpar.2009.12.001 20036058

[29] Rode B Bavdek SV Lackovic G Fazarinc G Bidovec A. Immunohistochemical study of normal and mange (S. scabiei var rupicaprae) infested chamois (Rupicapra rupicapra L) skin. Anat Histol Embryol. (1998) 27:187–92. 10.1111/j.1439-0264.1998.tb00178.x 9652147

[30] Salvadori C Finlayson J Trogu T Formenti N Lanfranchi P Citterio C. Characterization of immune system cell subsets in fixed tissues from alpine chamois (*Rupicapra rupicapra*). J Compar Pathol. (2016) 155:207–12. 10.1016/j.jcpa.2016.06.012 27476109

[31] Kumar V Abbas AK Aster JC. (editors). Diseases of the immune system. In: Robbins and Cotran Pathologic Basis of Disease. 9th edn. Philadelphia, PA: Elsevier (2015). p. 200–11.

[32] Hargis AM Myers S The integument. In: Zacary JF, editor. Pathologic Basis of Veterinary Disease. St. Louis: Elsevier (2017). p.1040–2.

[33] Bornstein S Zakrisson G Humoral antibody response to experimental Sarcoptes scabiei var. vulpes infection in the dog. Vet Dermatol. (1993) 4:107–10. 10.1111/j.1365-3164.1993.tb00202.x PMC8095404 8669378

[34] Arlian LG Morgan MS Rapp CM Vyszenski-Moher DL. The development of protective immunity in canine scabies. Vet Parasitol. (1996) 62:133–42. 10.1016/0304-4017(95)00854-3 8638386

[35] Lower KS Medleau LM Hnilica K. Evaluation of an enzyme-linked immunosorbent assay (ELISA) for the serological diagnosis of sarcoptic mange in dogs. Vet Dermatol. (2001) 12:315–20. 10.1046/j.0959-4493.2001.00265.x 11844220

[36] Scott DW Miller WH Griffin CE Muller & Kirk's Small Animal Dermatology, 6th ed. Philadelphia, PA: W.B. Saunders (2001). p. 476–83.

[37] Walton SF. The immunology of susceptibility and resistance to scabies. Parasite Immunol. (2010) 32:532–40. 10.1111/j.1365-3024.2010.01218.x 20626808

[38] Gross TL Ihrke PJ Walder EJ Affolter VK. (editors). Perivascular diseases of the dermis. In: Skin Diseases of the Dog and Cat: Clinical and Histopathologic Diagnosis. 2nd edn. Oxford: Blackwell Publishing (2005). p. 217.

[39] Partel P Citterio CV Cavallero S *Alpine ibex* (Capra ibex) versus *Sarcoptes scabiei*: field evidence of possible resistance as a driver for management and research. Hystrix. (2016) 27(ATIt Congress Supplement):21 10.4404/hystrix-27.0-11877

[40] Salvadori C Rocchigiani G Lazzarotti C Formenti N Trogu T Lanfranchi P. Histological Lesions and Cellular Response in the Skin of Alpine Chamois (Rupicapra r. rupicapra) Spontaneously Affected by Sarcoptic Mange. London: Hindawi Publishing Corporation, BioMed Research International (2016). p. 1–8. 10.1155/2016/3575468 PMC4925969 27403422

[41] Skerratt LF. Cellular response in the dermis of common wombats (Vombatus ursinus) infected with Sarcoptes scabiei var. wombat. J Wildl Dis. (2003) 39:193–202. 10.7589/0090-3558-39.1.193 12685083

[42] Berger A. Th1 and Th2 responses: what are they? BMJ. (2000) 321:424. 10.1136/bmj.321.7258.424 10938051 PMC27457

[43] Nimmervoll H Hoby S Robert N Lommano E Welle M Ryser-Degiorgis MP. Pathology of sarcoptic mange in red foxes (vulpes vulpes): macroscopic and histologic characterization of three disease stages. J Wildl Dis. (2013) 49:91–102. 10.7589/2010-11-316 23307375

[44] Oleaga A Casais R Prieto JM Gortázar C Balseiro A Comparative pathological and immunohistochemical features of sarcoptic mange in five sympatric wildlife species in Northern Spain. Eur J Wildl Res. (2012) 58:997–1000. 10.1007/s10344-012-0662-y

[45] Oleaga A Casais R Balseiro A Esp í A Llaneza L Hartasánchez A. (2011). New techniques for an old disease: sarcoptic mange in the Iberian wolf. Vet. Parasitol. 181, 255–266. 10.1016/j.vetpar.2011.04.036 21600696

[46] Alasaad S Granados JE Fandos P Cano-Manuel FJ Soriguer RC Pérez JM. The use of radio-collars for monitoring wildlife diseases: a case study from Iberian ibex affected by Sarcoptes scabiei in Sierra Nevada, Spain. Paras Vect. (2013) 6:242. 10.1186/1756-3305-6-242 23965311 PMC3765276

[47] Stone SP Scabies and pediculosis. In: Freedberg IM, et al., editors. Fitzpatrick's Dermatology in General Medicine, 6th edn. New York, McGraw-Hill (2003). p. 2283–9.

[48] Morris DO Dunstan RW. A histomorphological study of sarcoptic acariasis in the dog: 19 cases. J Am Anim Hosp Assoc. (1996) 32:119–24. 10.5326/15473317-32-2-119 8680917

[49] Mikuloaska A Falck B Distributional changes of Langerhans cells in human skin during irritant contact dermatitis. Arch Dermatol Res. (1994) 286:429–33. 10.1007/BF00371567 7532388

[50] Gurish MF Lynch DH Yowell R Daynes RA. Abrogation of epidermal antigen-presenting cell function by ultraviolet radiation administered in vivo. Transplantation. (1983) 36:304–9. 10.1097/00007890-198309000-00015 6612766

[51] Rico MJ Streilen JW. Comparison of alloimmunogenicity of Langerhans cells and keratinocytes from mouse epidermis. J Invest Dermatol. (1987) 89:607–10. 10.1111/1523-1747.ep12461373 3316412

[52] Schweizer J. Langerhans cell-free regions in orthokeratinizing sole-of-foot epidermis of adult mouse. Arch Dermatol Res. (1980) 268:157–60. 10.1007/BF00403800 6448586

[53] Cavallero S Citterio C Lanfranchi P Rossi L Obber F D'Amelio S Host-pathogen interaction in modulating variability at MHC DRB1 locus in alpine chamois. In: Proceeding of the II Rupicapra Symposium. Biology, Health, Monitoring and Management. Catalonia: Bellver de Cerdanya (2013). p. 24–25.

[54] Luzzago C Ebranati E Cabezón O Fernández-Sirera L Lavín S Rosell R. Spatial and temporal phylogeny of border disease virus in pyrenean chamois (Rupicapra p. pyrenaica). PLoS ONE. (2016) 11:e0168232. 10.1371/journal.pone.0168232 28033381 PMC5199066

[55] Alasaad S Rossi L Heukelbach J Pérez JM Hamarsheh O Otiende M. The neglected navigating web of the incomprehensibly emerging and re-emerging Sarcoptes mite. Infect Genet Evol. (2013) 17:253–9. 10.1016/j.meegid.2013.04.018 23624188

[56] Alasaad S Walton S Rossi L Bornstein S Abu-Madi M Soriguer R. Sarcoptes-world molecular network (Sarcoptes-WMN): integrating research on scabies. Int J Infect Dis. (2011) 15:e294–7. 10.1016/j.ijid.2011.01.012 21454116

